# An elderly man with pulmonary hypertension

**DOI:** 10.4103/1817-1737.74277

**Published:** 2011

**Authors:** Prashanth Panduranga, Mohammed Mukhaini

**Affiliations:** *Department of Cardiology, Royal Hospital, Muscat - 111, Sultanate of Oman*

A 70-year-old male, non-smoker with no past medical or surgical problems presented with two months history of worsening exertional dyspnea. He denied any history of chest pain, cough or hemoptysis. A transthoracic echocardiogram (TTE) done in a regional hospital showed a large atrial septal defect with severe pulmonary hypertension (PH) and was referred to our center for transesophageal echocardiogram (TEE) and possible device closure or surgery. Physical examination revealed a moderately built male without any distress. There was no cyanosis or clubbing. Cardiac examination revealed jugular venous pulse 4 cm above the sternal angle with prominent a-wave, right parasternal heave, loud P2, 2/6 pansystolic murmur at the left sternal border, and no pedal edema. Respiratory examination was normal. ECG showed sinus rhythm, right axis deviation and right ventricular hypertrophy. Chest radiograph was suggestive of mild cardiomegaly with no prominence of pulmonary vessels and clear lungs.

TTE and TEE were done [Figures 1 and 2].

**Figure 1:**
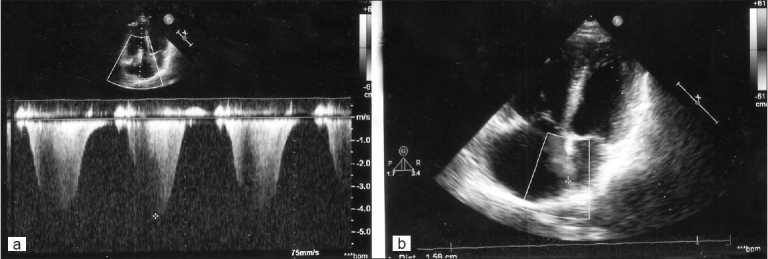
(a) Continuous wave Doppler echocardiography at tricuspid valve. (b) Transthoracic echocardiography four-chamber view

**Figure 2:**
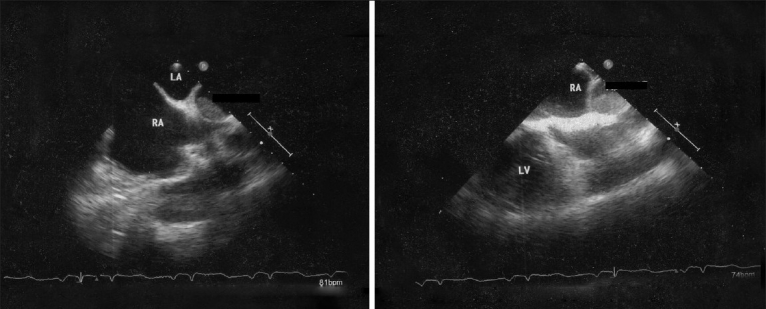
Transesophageal echocardiography in modified short-axis views. LA=Left atrium; RA=Right atrium; LV=Left ventricle

## Questions

1. What are the findings by TTE?
2. What is seen by TEE?
3. What is seen on computed tomography (CT) scan of chest (arrowheads)? What additional investigations are needed?
4. What is the diagnosis in this patient?

## Answers

**Figure 3:**
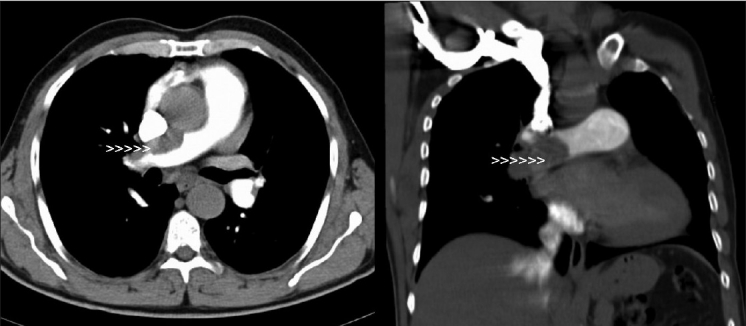
CT scan of chest in axial and coronal planes

1. A transthoracic echocardiogram (TTE) showed a markedly dilated right side with leftward septal bulging and moderate tricuspid regurgitation with calculated PA systolic pressure of 80 mmHg [Figure 1a]. Tricuspid annular plane systolic excursion was reduced at 1.6 cm (normal ≥2.0 cm). Pulmonary arteries (PA) were non-dilated and there was no intracardiac clot seen with good left ventricular systolic and diastolic function. Additionally, there was an echo dropout in the mid-interatrial septum [Figure 1b].
2. A transesophageal echocardiogram (TEE) done showed intact atrial septum even on contrast study. Previous TTE had demonstrated a false echo dropout. There was neither an atrial septal defect nor patent foramen ovale seen. A comprehensive TEE examination was done to find any cause. Incidentally, there was an echogenic mass seen in the right PA suggestive of organized pulmonary thrombus [Figure 2].
3. A computed tomography (CT) scan of chest confirmed proximal right PA thrombus causing partial obstruction [Figure 3]. Doppler ultrasound of both lower extremities showed no evidence of venous thrombosis. His blood investigations were normal including autoimmune and thrombophilia profile.
4. A diagnosis of chronic thromboembolic pulmonary hypertension (CTEPH) was made. He was treated with oral anticoagulation for three months, but there was persistence of PA thrombus and PH. He was advised to undergo pulmonary endarterectomy, but he declined.

## Discussion

A chronic thromboembolic pulmonary hypertension (CTEPH) is characterized by unresolved organized thrombus within the pulmonary arteries. Initial autopsy studies estimated the incidence of CTEPH at 0.1–0.5% in patients surviving acute pulmonary embolism (PE).[1] Recent prospective studiesindicate that CTEPH is more common than previously thought with an incidence ranging from 1.5 to 3.8% following acute symptomatic idiopathic PE.[2,3] However, CTEPH may occur in the absence of a clear history of acute PE in more than 50% of the cases.[1]

This case emphasizes the importance of a thorough echocardiographic examination in patients with pulmonary hypertension. In practice, CTEPH is often mistaken for chronic obstructive pulmonary disease, interstitial lung disease, asthma, atrial septal defect, obesity, deconditioning, or hyperventilation syndrome because of its rarity. Many patients with CTEPH present late in the course of disease with progressive dyspnea on exertion, exercise intolerance, hemoptysis, signs of PH and right heart failure. Nonspecific symptoms and lack of medical history of previous venous thromboembolism often complicate accurate diagnosis and, as a result, CTEPH is frequently misdiagnosed and is under recognized in practice.[1]

The majority of patients with CTEPH have no predisposing factors; however, in a recent study, ventriculo-atrial shunts, infected pacemakers, splenectomy, previous or recurrent venous thromboembolism, blood groups other than O, lupus anticoagulant/antiphospholipid antibodies, were more often associated with CTEPH.[4] Thyroid replacement therapy and, history of malignancy emerged as novel CTEPH risk factors. Although organized central thrombi are the most likely disease-initiating event, progressive small pulmonary vessel arteriopathy may contribute to the long-term progression of PH.[1]

The diagnosis is usually made by ventilation-perfusion scan and/or CT pulmonary angiogram. However, TTE should be the initial investigation to confirm PH and rule out other causes. TEE should be considered to exclude shunts, as in this patient. We emphasize that, in patients with undiagnosed exertional dyspnea following TTE, a ventilation-perfusion scan and/or CT pulmonary angiogram should be done to exclude CTEPH. Right heart catheterization and pulmonary arteriography is needed pre-operatively to determine the feasibility of endarterectomy according to the location of the disease, proximal versus distal. Recently, CT is being established as alternative to conventional angiography, not only for diagnosing chronic pulmonary thromboembolism, but also for determining which cases are treatable with surgery and confirming technical success postoperatively.[5]

Pulmonary endarterectomy is the first treatment option, followed by chronic oral anticoagulation in inoperable patients.[6] Pulmonary endarterectomy may restore hemodynamics in 80% of cases.[7] Other medical treatment options for persistence PH include prostacyclin analogs(epoprostenol), endothelin receptor antagonists (bosentan), or phosphodiesterase-5 inhibitors (sildenafil). In a recent study, comparing surgery and medical therapy (pulmonary vasodilator therapy), one- and three-year survival from diagnosis was 82 and 70% for patients with nonsurgical disease and 88 and 76% for those treated surgically.[8] CTEPH has emerged as a ‘dual’ pulmonary vascular disorder with major vessel vascular remodeling of thrombus organization, combined with a small vessel pulmonary arteriopathy that is a target for classic vasodilator treatments.[7] In the absence of severe comorbidity, lung transplantation may be undertaken where pulmonary endarterectomy has failed, in nonresponders to medical therapy, and in patients with progressive arteriopathy.[6]

In conclusion, this case illustrates that CTEPH may be misdiagnosed and is under-recognized in clinical practice, and a high index of suspicion is required to diagnose this potentially treatable disease. In addition, this case demonstrates the usefulness of TEE in diagnosing CTEPH.
